# Pretransplant Serum Creatinine in Peritoneal Dialysis Patients Predicts Graft Outcomes

**DOI:** 10.1016/j.xkme.2025.101056

**Published:** 2025-06-24

**Authors:** Faisal Jarrar, Karthik K. Tennankore, Ngan N. Lam, David A. Clark, Bryce A. Kiberd, Amanda J. Vinson

**Affiliations:** 1 Faculty of Medicine, University of Calgary, Calgary, Alberta, Canada; 2 Division of Nephrology, Department of Medicine, Dalhousie University, Halifax, Nova Scotia, Canada; 3 Nova Scotia Health, Halifax, Nova Scotia, Canada; 4 Cumming School of Medicine, University of Calgary, Calgary, Alberta, Canada

**Keywords:** Creatinine, eGFR, home dialysis, kidney, peritoneal dialysis, transplant

## Abstract

**Rationale & Objective:**

Although peritoneal dialysis (PD) as a pretransplant dialysis modality is associated with favorable outcomes after kidney transplant, it is unknown if pretransplant serum creatinine (Scr) level is associated with subsequent graft outcomes in candidates managed with PD. Our objective was to examine the association between Scr at the time of transplant and short-term and long-term outcomes posttransplant.

**Study Design:**

Retrospective cohort study.

**Setting & Participants:**

A total of 20,166 adult (≥18 years of age) patients who were receiving PD at the time of a first living or deceased donor kidney transplant in the United States between 2000 and 2017, identified using the Scientific Registry of Transplant Recipients database.

**Exposures:**

Primary exposure was final Scr level before transplant, categorized as <5, 5-8, 8-12, and >12 mg/dL. Sensitivity analyses for patient subgroups included recipient age (≥50 vs <50 years) and dialysis vintage (≥3 vs <3 years) at transplant.

**Outcomes:**

The primary outcome was death-censored graft loss (DCGL). Secondary outcomes included all-cause graft loss and delayed graft function (DGF).

**Results:**

Pretransplant Scr was significantly associated with DCGL (adjusted HR, 1.17; 95% CI, 1.02-1.34 for Scr >12 mg/dL [reference <5 mg/dL]) and DGF (adjusted OR, 2.71; 95% CI, 2.26-3.26 for Scr >12 mg/dL [reference <5 mg/dL]). There was no association with all-cause graft loss. The risk of DCGL and DGF associated with high pretransplant Scr was higher for those who were older (≥50 years) and those with longer dialysis vintage (≥3 years).

**Limitations:**

No access to potential predictors of pretransplant Scr including residual kidney function, dialysis adequacy and adherence; exact timing of Scr values pretransplant was unknown.

**Conclusions:**

To our knowledge, this is the first study to explore the association between pretransplant Scr level in PD patients and graft outcomes after kidney transplantation. The reason for this increased risk is unclear but may reflect reduced residual kidney function, among other factors.

## Plain-Language Summary

Kidney transplantation is a life-saving procedure and is associated with improved survival for people living with kidney failure. However, kidney transplants may fail during the lifetime of transplant recipients. Identifying treatable factors that contribute to kidney transplant failure before transplantation is important. Many of these factors relate to dialysis before transplantation. Therefore, our study examined whether creatinine level before transplantation in peritoneal dialysis patients influences transplant survival. Our study of more than 20,000 patients from the United States found that peritoneal dialysis patients with elevated pretransplant creatinine levels have a significantly higher risk of transplant loss. This finding is important because it identifies high creatinine as a predictor and potentially treatable factor that contributes to kidney transplant failure.

For patients with kidney failure, kidney transplantation is the preferred treatment option because it has been shown to improve survival as well as quality of life relative to remaining on dialysis.^1^ In the United States, only 2.9% of patients with new kidney failure receive a preemptive kidney transplant because of lack of suitable kidney donors, late referral to nephrology, or ongoing health and/or financial barriers.^2^ Pretransplant dialysis is therefore a common treatment option during the transition to transplant. The influence of pretransplant dialysis modality and dialysis-related factors on posttransplant graft outcomes is an important area of study and can offer insights for prediction and prevention of graft failure.^3^

In patients undergoing kidney transplant, those on peritoneal dialysis (PD) compared with hemodialysis as a pretransplant modality have been shown to have greater survival and reduced incidence of delayed graft function (DGF).^4^, ^5^, ^6^ Although the reasons for this association are not well established, PD may provide better preservation of native residual kidney function (RKF) than hemodialysis,^7^, ^8^, ^9^, ^10^, ^11^ which may favorably influence DGF and survival.^4^^,^^5^ Among a myriad of methods available for estimating RKF in PD patients, creatinine-based measurements have been proposed as a simple assessment or proxy of RKF.^12^ Total creatinine clearance (ie, peritoneal dialytic and renal creatinine clearance) is commonly used for RKF measurement and is recommended by current guidelines.^13^^,^^14^ Serum creatinine (Scr) level in PD patients may serve as a crude surrogate of creatinine clearance, and in turn, RKF. Higher Scr in PD patients may correlate with reduced RKF or greater patient size and/or metabolic demand, and this may associate with increased risk posttransplant. Conversely, lower pretransplant Scr in PD patients may reflect low muscle mass and/or protein-energy wasting, which may also associate with adverse posttransplant outcomes.^15^^,^^16^ The association between final creatinine in PD patients before transplant and subsequent graft outcomes may offer important insights for clinicians when risk stratifying patients pretransplant. To our knowledge, this association has not been examined. Therefore, in a population of patients receiving PD before kidney transplantation, we aimed to examine the association between Scr at the time of transplant and short-term and long-term outcomes posttransplant.

## Methods

### Subject Selection

We conducted a cohort study of adult (≥18 years of age) patients who were receiving PD at the time of a first living or deceased donor kidney transplant in the United States between January 1, 2000, and December 31, 2016, identified using the Scientific Registry of Transplant Recipients (SRTR) database. We excluded patients aged <18 years, those receiving hemodialysis at the time of transplant, preemptive transplant recipients, and those receiving multiple organs, en bloc, or sequential transplants.

### Exposure

The primary exposure was recipient Scr level at the time of transplant (collected most proximal to the time of transplant). Scr values were categorized into 4 groups a priori to achieve a roughly even group distribution: <5, 5-8, 8-12, and >12 mg/dL.

### Outcomes

The primary outcome was death-censored graft loss (DCGL), defined as return to maintenance dialysis or preemptive repeat transplantation. Secondary outcomes included the composite of graft failure or death (ie, all-cause graft loss [ACGL]) and DGF, defined as receipt of dialysis within the first 7 days posttransplant. Censoring occurred at loss to follow up and at the date of last follow-up.

### Data Collection and Covariates

Known literature predictors of graft loss including donor and recipient age and sex, donor-recipient weight ratio, recipient body mass index (BMI), cause of recipient kidney failure, prior transplant status, donor type (living vs deceased), donation after circulatory death status, human leukocyte antigen mismatches, peak panel reactive antibody, and recipient type 2 diabetes were selected a priori as variables for inclusion in multivariable models. Missing data were treated by case-wise deletion.

### Analysis

Descriptive statistics are reported for baseline characteristics. Means and standard deviations and medians and interquartile ranges were used for continuous normal and continuous nonnormally distributed variables.

#### Primary Analysis

We used a multivariable Cox proportional hazards model to determine the adjusted hazard ratio (aHR) for DCGL according to recipient pretransplant Scr (relative to a pretransplant creatinine of <5 mg/dL), adjusted for known predictors of graft loss as listed above. Differences in survival were visually displayed using the Kaplan-Meier product limit method, and differences were statistically compared using the log-rank test.

#### Secondary Analyses

A multivariable Cox proportional hazards model was used to examine the aHR of ACGL for each category of pretransplant creatinine, as above. The adjusted odds ratio of DGF for each category of pretransplant candidate creatinine was examined using multivariable logistic regression.

#### Sensitivity Analyses

Sensitivity analyses were conducted to better characterize the association between pretransplant Scr and each outcome. These included stratifying by (1) dialysis vintage (dichotomized at 3 years), considering longer dialysis vintage is known to affect RKF^17^; (2) recipient age (dichotomized at 50 years), considering the known association between age and creatinine^18^; (3) recipient median BMI; and (4) quartile of Kidney Donor Risk Index (KDRI).

An additional sensitivity analysis repeated the primary analysis using an exposure of recipient estimated glomerular filtration rate (eGFR) rather than pretransplant creatinine. The 2021 race-free CKD-EPI (Chronic Kidney Disease Epidemiology Collaboration) creatinine equation was used to derive eGFR values based on the available Scr value, acknowledging that contributions from PD clearance influence Scr and the measured eGFR value is a representation of RKF and muscle mass (as for patients not receiving dialysis) but also the additional dialysis-mediated clearance.^19^ eGFR was categorized into 4 groups a priori: <4, 4-8, 8-12, and >12 mL/min/1.73 m^2^. An eGFR >12 mL/min/1.73 m^2^ was considered the reference category for this analysis. Finally, given the potential association between elevated pretransplant creatinine and early graft loss, we repeated the primary analysis after excluding patients with early graft failure (ie, <90-day graft survival).

This study used data from the SRTR. The SRTR data system includes data on all donors, waitlisted candidates, and transplant recipients in the United States, submitted by the members of the Organ Procurement and Transplantation Network. The Health Resources and Services Administration, US Department of Health and Human Services provides oversight to the activities of the Organ Procurement and Transplantation Network and SRTR contractors. The data reported here were supplied by the Hennepin Healthcare Research Institute as the contractor for the SRTR. The interpretation and reporting of these data are the responsibility of the authors and in no way should be seen as an official policy of or interpretation by the SRTR or the US Government.

The study protocol was submitted to the Nova Scotia Health Research Ethics Board who deemed the study met requirements outlined in the Tri-Council Policy Statement for an REB review exemption (REB FILE #: 1029917). Informed consent was waived because of the use of deidentified information, as is standard practice for the SRTR. All statistical analyses were performed using Stata version 13.1 (Stata Corp). For statistical comparisons, *P* < 0.05 was deemed the threshold for statistical significance.

## Results

### Baseline Characteristics

**Figure 1:**
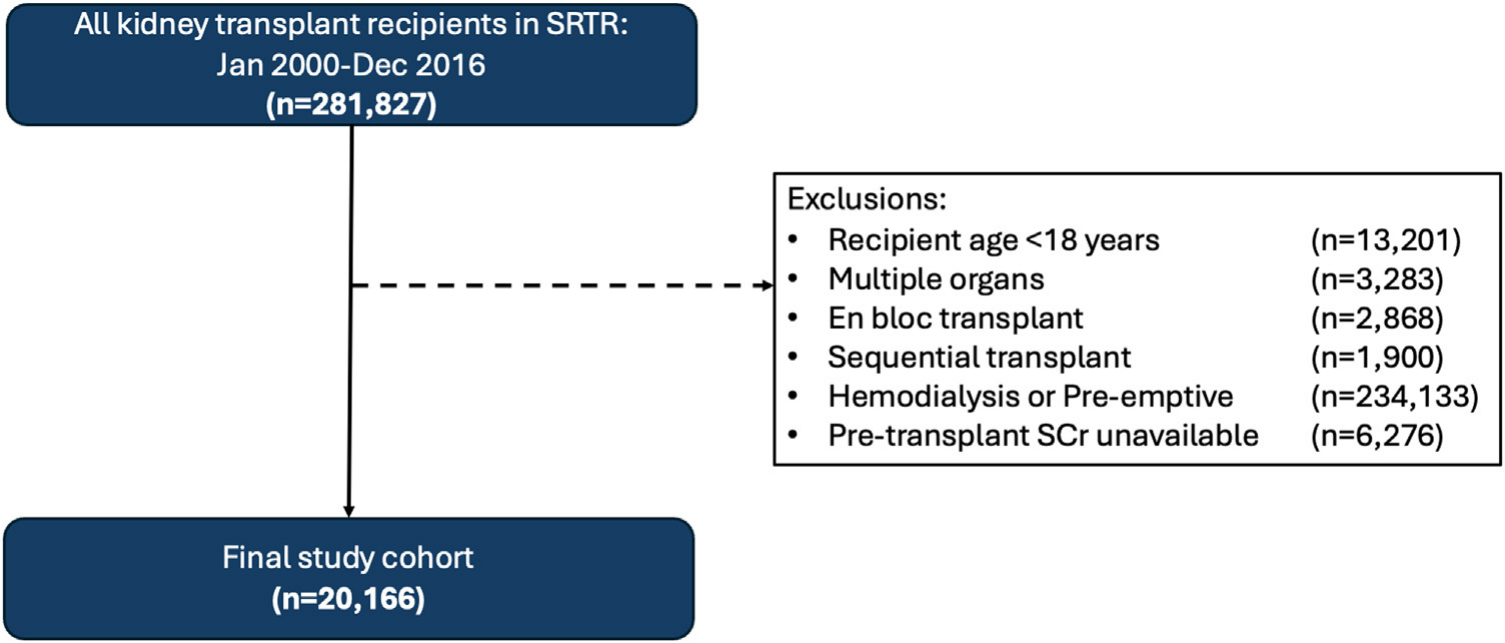
Final study cohort after exclusions. Scr, serum creatinine; SRTR, Scientific Registry of Transplant Recipients.

After relevant exclusions, our final study cohort consisted of 20,166 patients (Fig 1). Baseline characteristics are shown in Table 1. The majority of transplanted kidneys (n = 13,013 [65.5%]) were from deceased donors, and recipient mean dialysis vintage was 3.4 years (standard deviation, 2.7 years). Among all recipients, pretransplant median Scr was 8.3 mg/dL (5.9-11.7 mg/dL) and median eGFR was 6.4 mL/min/1.73 m^2^ (4.3-9.7 mL/min/1.73 m^2^). DCGL occurred in 3,563 patients (17.7%), ACGL in 6,950 (34.5%), and DGF in 2,862 (14.2%). Median time to DCGL was 3.9 years (interquartile range, 1.6-6.8 years) and median time to ACGL was 4.0 years (interquartile range, 1.6-7.0 years).

**Table 1:** Baseline Characteristics of Recipients Included in the Final Analysis

Characteristics	All recipients N = 20,166
Donor age, y	39 (27-50)
Recipient age, y	49 (38-59)
Female recipient	8,968 (44.4%)
Pretransplant Scr, mg/dL	
<5	3,120 (15.5%)
5-8	6,312 (31.3%)
8-12	5,958 (29.5%)
>12	4,776 (23.7%)
Pretransplant eGFR, mL/min/1.73 m^2^	
<4	4,083 (20.2%)
4-8	8,726 (43.3%)
8-12	4,347 (21.6%)
>12	3,010 (14.9%)
Recipient race	
White	14,194 (70.4%)
Black	4,369 (21.7%)
Other	1,602 (7.9%)
Recipient BMI, kg/m^2^	
<18	494 (2.6%)
18-25	6,065 (32.0%)
25-30	6,434 (34.0%)
30-35	4,091 (21.6%)
>35	1,844 (9.74%)
Dialysis vintage, y	
<3	9,354 (54.8%)
>3	7,715 (45.2%)
Living donor	7,153 (35.5%)
Dialysis vintage, y	2.73 (1.52-4.46)
Cold ischemia time, h	12 (2.4-20)
Human leukocyte antigen mismatch	
0	1,879 (9.3%)
1	734 (3.6%)
2	1,840 (9.1%)
3	3,707 (18.4%)
4	4,390 (21.8%)
5	5,049 (25.0%)
6	2,507 (12.4%)
Panel reactive antibody	
<20%	14,212 (81.6%)
20%-80%	2,125 (12.3%)
>80%	877 (5.1%)
Previous transplant	2190 (10.9%)
Recipient comorbid conditions	
Type 2 diabetes	5,475 (27%)
Hypertension	17,812 (88.3%)
Coronary artery disease	1,548 (8.1%)
Peripheral vascular disease	910 (4.5%)
Cause of kidney failure	
Diabetes	4,443 (22.0%)
Glomerulonephritis	6,269 (31.1%)
Polycystic kidney disease	1,466 (7.3%)
Hypertension	4,426 (21.9%)
Other	2,729 (13.5%)

*Note:* Values are n (%) or median (interquartile range). Proportion missing: recipient BMI (6.1%), donor BMI (2.2%), human leukocyte antigen mismatch (0.3%); preemptive (2.6%); recipient diabetes (0.3%); recipient hypertension (2.4%); recipient coronary artery disease (5.2%); recipient peripheral vascular disease (2.4%); end-stage renal disease (4.1%); panel reactive antibody (14.6%); preemptive (0.5%), dialysis vintage (15.4%), cold ischemia time(11.3%).

Abbreviations: BMI, body mass index; eGFR, estimated glomerular filtration rate; Scr, serum creatinine.

### Association Between Recipient Pretransplant Scr and DCGL

**Figure 2:**
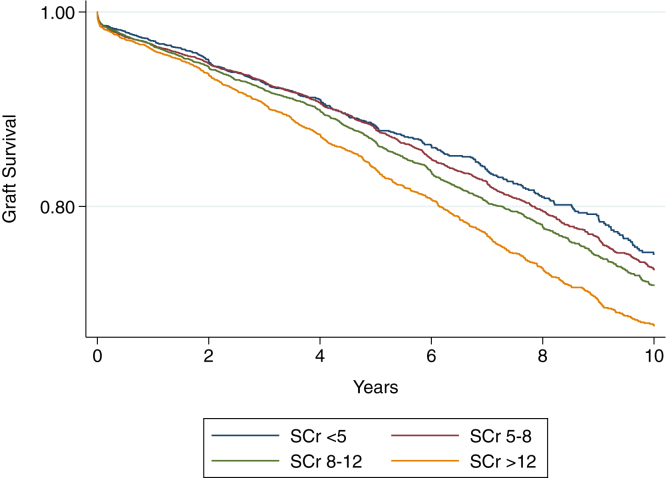
Kaplan-Meier curves demonstrating time to death-censored graft loss for recipient pretransplant Scr (mg/dL). Log-rank *P* < 0.001. Scr, serum creatinine.

Recipient pretransplant Scr was significantly associated with DCGL at the highest creatinine category (aHR, 1.17; 95% CI, 1.02-1.34 for Scr >12 mg/dL relative to <5 mg/dL), with no significant association observed for any other pretransplant creatinine threshold (Table 2). Kaplan-Meier survival curves demonstrating time to DCGL by categories of pretransplant Scr are depicted in Fig 2, log-rank *P* < 0.001.

**Table 2:** Adjusted Risk of Graft Outcomes for Recipient Pretransplant Serum Creatinine

	Pretransplant Scr (mg/dL)		
	5-8 (n = 6,312)	8-12 (n = 5,958)	>12 (n = 4,776)
Death-censored graft loss HR (95% CI)	1.03 (0.91-1.17)	1.08 (0.95-1.23)	1.17 (1.02-1.34)^a^
All-cause graft loss HR (95% CI)	0.94 (0.87-1.02)	0.95 (0.87-1.03)	1.02 (0.93-1.12)
Delayed graft function OR (95% CI)	1.43 (1.20-1.71)^a^	1.95 (1.63-2.32)^a^	2.71 (2.26-3.26)^a^

*Note:* Reference category was pretransplant Scr <5 mg/dL. Adjusted for recipient body mass index, recipient age, recipient sex, donor sex, donor age, donor-recipient weight ratio, donation after circulatory death status, human leukocyte antigen mismatch, recipient diabetes, panel reactive antibody group, donor type, cause of kidney failure, and previous kidney transplant.

Abbreviations: CI, confidence interval; HR, hazard ratio; OR, odds ratio; Scr, serum creatinine.

a Statistically significant.

### Association Between Recipient Pretransplant Scr and Secondary Outcomes

Pretransplant Scr was not significantly associated with risk of ACGL; however, there was a stepwise increased risk of DGF with increasing pretransplant creatinine (Table 2). Relative to a pretransplant Scr <5 mg/dL, the highest risk of DGF was observed for a pretransplant Scr >12 mg/dL (odds ratio, 2.71; 95% CI, 2.26-3.26), whereas the lowest risk (although still significant) was observed for a pretransplant Scr of 5-8 mg/dL (odds ratio, 1.43; 95% CI, 1.20-1.71).

### Sensitivity Analyses

#### Dialysis Vintage

The association between pretransplant Scr and graft outcomes after stratifying by dialysis vintage is shown in Table 3. A pretransplant Scr was only associated with DCGL (aHR, 1.44; 95% CI, 1.08-1.90 for Scr >12 mg/dL) in recipients with longer (≥3 years) but not shorter dialysis vintage (Table 3). SCr was not associated with ACGL regardless of dialysis vintage; the association with DGF was stronger for those with longer dialysis vintage.

**Table 3:** Adjusted Risk of Death-censored Graft Loss for Recipient Pretransplant Scr Stratified by Dialysis Vintage

	Pretransplant Scr (mg/dL)					
	5-8		8-12		>12	
	Vintage <3 y (n = 3,380)	Vintage ≥3 y (n = 1,959)	Vintage <3 y (n = 2,667)	Vintage ≥3 y (n = 2,354)	Vintage <3 y (n = 1,646)	Vintage ≥3 y (n = 2,523)
Death-censored graft loss HR (95% CI)	0.97 (0.81-1.15)	1.30 (0.97-1.73)	1.03 (0.86-1.23)	1.34^a^ (1.01-1.77)	1.15 (0.94-1.40)	1.44^a^ (1.08-1.90)
All-cause graft loss HR (95% CI)	0.89 (0.79-0.99)	1.09 (0.91-1.29)	0.90 (0.80-1.02)	1.09 (0.92-1.30)	0.97 (0.84-1.11)	1.16 (0.97-1.38)
Delayed graft function OR (95% CI)	1.15 (0.87-1.48)	1.73^a^ (1.31-2.30)	1.79^a^ (1.38-2.32)	1.80^a^ (1.36-2.37)	2.22^a^ (1.66-2.97)	2.59^a^ (1.95-3.43)

*Note:* Reference category was pretransplant Scr <5 mg/dL. Adjusted for recipient body mass index, recipient age, recipient sex, donor sex, donor age, donor-recipient weight ratio, donation after circulatory death status, human leukocyte antigen mismatch, recipient diabetes, panel reactive antibody group, donor type, cause of kidney failure and previous kidney transplant.

Abbreviations: CI, confidence interval; HR, hazard ratio; OR, odds ratio; Scr, serum creatinine.

a Statistically significant.

#### Recipient Age

The association between pretransplant Scr and graft outcomes after stratifying by recipient age is shown in Table S1. Pretransplant Scr level was not associated with DCGL in younger recipients (age <50 years); however, there was a significantly increased risk with an elevated pretransplant Scr in older (≥50 years) recipients (aHR, 1.26; 95% CI, 1.01-1.56 for Scr >12 compared with Scr <5 mg/dL). Scr was more strongly associated with DGF in older patients (≥50 years) for all creatinine ranges with no association seen for ACGL for any of the creatinine groups at either age cutoffs.

#### Recipient BMI and KDRI

Pretransplant Scr was only associated with risk of DCGL in recipients with BMI greater than the median (aHR, 1.26; 95% CI, 1.04-1.53 for Scr >12 mg/dL; Table S2) and in those receiving donor kidneys in the highest KDRI quartile (aHR, 1.36; 95% CI, 1.01-1.84 for Scr >12 mg/dL; Table S3).

#### eGFR

**Figure 3:**
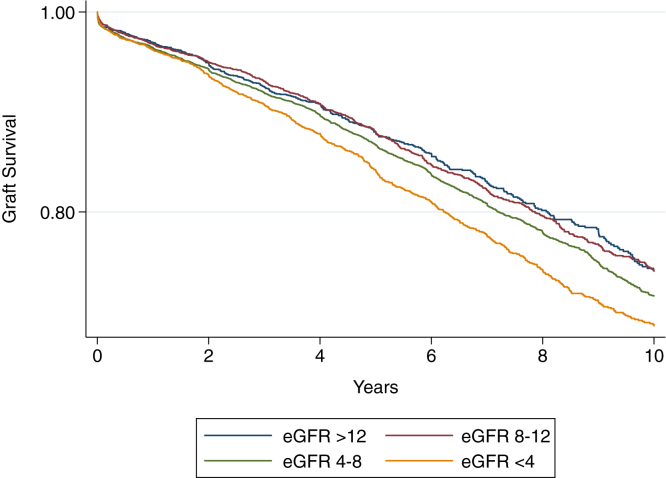
Kaplan-Meier curves demonstrating time to death-censored graft loss for recipient pretransplant eGFR (mL/min/1.73 m^2^). Log-rank *P* < 0.001. eGFR, glomerular filtration rate.

There was an increased risk of DCGL with lower pretransplant eGFR. Relative to recipients with an eGFR >12 mL/min/1.73 m^2^ pretransplant, the lowest eGFR group (<4 mL/min/1.73m^2^) was associated with the highest risk of DCGL (aHR, 1.18; 95% CI, 1.03-1.35); no association was seen for eGFR 4-8 and eGFR of 8-12 mL/min/1.73 m^2^ (Table S4). The Kaplan-Meier survival curves for categories of pretransplant eGFR and DCGL are shown in Fig 3. eGFR was not associated with risk of ACGL but did associate with a gradually increased risk of DGF as pretransplant eGFR decreased.

#### Early Graft Loss

The association between pretransplant Scr and DCGL excluding any patients with early graft loss is shown in Table S5. The results are similar to the primary analysis in which pretransplant Scr was significantly associated with DCGL in only the highest creatinine category (aHR, 1.19; 95% CI, 1.03-1.37 for creatinine >12 mg/dL relative to <5 mg/dL).

## Discussion

In this study, we show for the first time that a higher pretransplant Scr in patients receiving PD is associated with an increased risk of early and late complications post-kidney transplant, namely an increased risk of DCGL and DGF.

In patients treated with PD, Scr may be influenced by a number of competing factors, including RKF, patient size, metabolic demand, adherence with PD prescription, and PD clearance parameters.^14^^,^^15^^,^^20^ Creatinine clearance (ie, peritoneal dialytic and renal creatinine clearance) is commonly used as a proxy for RKF, which is well-recognized as an important marker of outcomes in PD.^14^ Greater RKF is associated with easy fluid management, improved nutrition, reduced erythropoietin requirements, improved potassium clearance, improved quality of life, and greater survival.^14^^,^^21^, ^22^, ^23^, ^24^, ^25^, ^26^ Although this requires further study, Scr in PD patients may be viewed as a proxy or estimate of total creatinine clearance. We show for the first time that a high pretransplant Scr is significantly associated with increased risk of DCGL and DGF.

PD patients with lower Scr relating to preserved RKF may share some similarities with preemptive kidney transplant recipients. It has been demonstrated that preemptive kidney transplant recipients who have sufficient RKF to remain off dialysis experience fewer complications than those established on dialysis at the time of transplant.^27^^,^^28^ Proposed reasons for this include better general health pretransplant, preventing the complications of prolonged uremia, and avoidance of dialysis-related risks including cardiovascular risk and immune dysfunction.^29^ PD patients with lower pretransplant Scr may be functionally more akin to preemptive transplant recipients than anuric patients maintained on hemodialysis or PD patients with waning RKF.

Aside from differences in RKF, pretransplant Scr in PD patients may correlate with dialysis adequacy or underdialysis, although this has not been previously tested. Although weekly Kt/V_urea_ or creatinine clearance remain the primary methods for measuring dialysis adequacy, creatinine-based assessments have been explored by some studies as a simple and inexpensive alternative for rapidly evaluating the adequacy of PD.^12^^,^^19^^,^^30^^,^^31^ Relative underdialysis would be expected to correlate with higher Scr in patients on PD (accounting for patient size, muscle mass, and other factors), although this has not been adequately explored in the literature. Poor dialysis adequacy is linked to a number of adverse effects, including volume overload, uremia, and cardiovascular risk.^32^, ^33^, ^34^ Moreover, a higher pretransplant Scr may indicate less adherence with PD prescriptions and dialysis recommendations, which would similarly contribute to poor outcomes. Adherence with immunosuppression medications has critical associations with risk of rejection and graft loss posttransplant,^35^, ^36^, ^37^ and whether higher Scr may be a predictor of prescription nonadherence in some patients is a point for future study. Overall, a higher pretransplant Scr may be a surrogate for a combination of risk factors that may negatively affect downstream graft survival.

Differences in body anthropometrics are also known to influence measured Scr, even among patients with normal kidney function; taller individuals with greater muscle mass typically have a higher baseline Scr level.^38^^,^^39^ Therefore, it is possible that high pretransplant creatinine may reflect differences in body stature, although our models were adjusted for BMI. It is well established that weight mismatch between kidney donors and recipients is associated with DCGL, with increased risk when the recipient is larger than the donor.^40^^,^^41^ A higher baseline Scr in patients receiving PD may indicate larger body mass and therefore a corresponding increased risk of DCGL due of potential size mismatch with the kidney donor. We adjusted for donor-recipient weight ratio in our analyses, which would have accounted for most of this effect, although it does not account for differences in muscle mass, lean body mass, or adiposity, all of which are differentially associated with metabolic demand and kidney hyperfiltration injury.^42^^,^^43^

In sensitivity analyses, the risk of elevated pretransplant creatinine was only evident among recipients with increased BMI and those receiving kidneys with the highest KDRI. The reasons for these findings require more investigation but may reflect the risk associated with high pretransplant Scr being potentiated in the setting of other risk factors (including recipient obesity^44^ and donor kidneys with higher KDRI^45^). There was no significant change to the signal of risk with increased pretransplant Scr when excluding patients with early graft loss, indicating that the mechanism of risk is not necessarily through increased surgical complications on account of uremia and uremic platelet bleeding dysfunction.

Interestingly, the risk of DCGL differed by pretransplant recipient age and dialysis vintage at transplant; higher Scr in PD patients at the time of transplant was associated with the greatest risk in older patients and those with longer dialysis vintage. Kidney function decreases with advancing age and nephron senescence^46^^,^^47^; therefore, high Scr in older individuals with waning muscle mass may be associated with correspondingly less RKF than the same creatinine level in a younger patient with greater muscle mass. The increased risk of high pretransplant creatinine observed among patients with longer dialysis vintage requires study. It is known that RKF decreases over time on dialysis,^17^ and therefore, the elevated creatinine levels in the longer dialysis vintage cohort may be more reflective of declining RKF rather than dialysis inadequacy or higher metabolic demand.

In addition to the increased risk of DCGL, we show a stepwise increased risk of DGF with higher pretransplant Scr values. The observed association between higher Scr and DGF may partly be explained by RKF, which is known to associate favorably with early posttransplant outcomes including DGF.^6^^,^^48^^,^^49^ Another potential significance of pretransplant Scr on early outcomes is its influence on clinician decision making. Patients with higher pretransplant Scr and slow graft function may be expected to maintain high Scr values posttransplant, which may influence providers to trigger dialysis more frequently than for patients with slow graft function starting with lower numerical creatinine values.

Elevated Scr in patients on PD at the time of kidney transplant was not associated with risk of ACGL. This finding may be explained by previously reported associations between Scr in dialysis patients and mortality.^16^^,^^50^^,^^51^ In maintenance hemodialysis patients, those with higher Scr values have been reported to have more favorable nutritional status, which likely reflects creatinine being a marker of muscle mass and protein-energy wasting.^52^ The association between Scr and nutritional status may have attenuated the risk of death and accounts for the differential associations seen for DCGL and ACGL, although the extent to which this risk persists after transplantation requires further study.

The associations described between pretransplant creatinine and graft outcomes were also observed for eGFR, whereby the lowest eGFR group was associated with the greatest risk of both DCGL and DGF, but not ACGL. Some studies have applied eGFR formulas to Scr values in PD patients to approximate creatinine clearances achieved by dialysis and reported good predictive ability.^19^ It is important to note that Scr and eGFR creatinine-based formulas have a number of limitations, including influence by muscle mass and body size and limited applicability to cirrhotic, paraplegic, malnourished, age <18 years, or, in general, participants in an unsteady metabolic state.^53^ However, there may be a rationale to using eGFR equations in the PD setting, because patients on PD are in a near-steady metabolic state, with relatively stable creatinine concentrations.^31^

Our study has implications for research. Although the pathophysiologic processes remain unclear, elevated Scr level pretransplant may emerge as a risk factor for adverse graft outcomes. To our knowledge, because this is the first study to report an association between pretransplant Scr and graft survival, additional studies are needed to examine whether our findings are reproducible, including in cohorts outside of North America. Furthermore, our study makes assumptions that Scr may be a proxy for factors such as RKF and dialysis adequacy. Future investigations should be targeted at examining whether these assumptions are valid in different patient populations and exploring the pathophysiologic mechanisms underlying the association between pretransplant Scr and graft outcomes. If further evidence emerges validating Scr as a risk factor in this domain, patients on PD with elevated Scr may warrant closer monitoring pretransplant or at least further justification by the clinician as to why their Scr is elevated. For reasons highlighted earlier in the discussion, Scr should be interpreted with caution in PD patients and likely has differential associations in different patient populations. For these reasons, we cannot advocate for transplant-related decisions to be made based on creatinine measurements, or for directed interventions that are aimed to lower it.

Although this study has important strengths, namely its novelty in examining pretransplant Scr and eGFR in a population of patients on PD, there are limitations. First, we did not have access to patients’ actual RKF, dialysis prescription, or adherence to dialysis before transplantation. These data would be interesting to include in a future analysis to determine the extent to which these factors correlate with pretransplant Scr/eGFR as well as graft outcomes. Second, the use of eGFR in dialysis patients is problematic.^54^ In a dialysis population, eGFR encompasses dialysis effects as opposed to true glomerular function. We therefore chose Scr as the primary exposure, and adjusted for age, sex, and body size in our multivariable model, which ultimately yielded similar results for both creatinine and eGFR. Third, we did not have data on the exact timing of Scr values pretransplant, and there may have been considerable variability between patients, although unlike hemodialysis patients, patients on PD typically express stability in Scr readings. Furthermore, we did not have access to other variables that may influence the association between Scr and graft outcomes, including frailty, serum albumin, and recipient socioeconomic factors. Finally, we did not have access to the indication for dialysis in patients with DGF, and this may have provided important insights to our results, especially if some patients were dialyzed based on creatinine thresholds in the absence of other acute indications. Nevertheless, although this would influence DGF, it would not be anticipated to affect the risk of long-term outcomes such as DCGL.

To our knowledge, this is the first study to explore the association between pretransplant Scr level and/or eGFR in PD patients and graft outcomes after kidney transplantation. We report that higher pretransplant creatinine is associated with risk of DCGL and DGF. Our findings highlight that final creatinine in PD patients before transplant may predict early and late graft outcomes and offer important insights to clinicians when risk stratifying patients pretransplant. Future studies may aim to more closely examine the pathophysiologic processes driving this association, including the relative contributions from RKF, dialysis adequacy, metabolic demand, and potentially adherence.
