# Supplementary material for: Pretransplant Serum Creatinine in Peritoneal Dialysis Patients Predicts Graft Outcomes
Source: Kidney Med. 2025 Jun 24;7(9):101056. doi: 10.1016/j.xkme.2025.101056 (PMC12332944; doi:10.1016/j.xkme.2025.101056)
Supplement: Supplementary File (PDF) — Tables S1-S6. [file mmc1.pdf]

Table S1: Adjusted risk of death-censored graft loss for recipient pre-transplant SCr (mg/dL), stratified by recipient age (≥50 and <50 years). Reference category used was pre-transplant SCr <5 mg/dL. *Bolded values are statistically significant.*

	Pre-transplant serum creatinine (mg/dL)					
	5-8		8-12		>12	
	Age <50 (n= 2,760)	Age ≥50 (n= 3,552)	Age <50 (n= 3,009)	Age ≥50 (n= 2,859)	Age <50 (n= 3,190)	Age ≥50 (n= 1,586)
Death-censored graft loss (aHR)	1.02 (0.85-1.21)	1.06 (0.88-1.28)	1.03 (0.87-1.22)	1.16 (0.96-1.41)	1.13 (0.95-1.35)	**1.26 (1.01-1.56)**
All-cause graft loss (aHR)	1.00 (0.86-1.15)	0.93 (0.84-1.03)	1.00 (0.87-1.16)	0.94 (0.84-1.04)	1.08 (0.93-1.25)	1.02 (0.90-1.16)
Delayed graft function (aOR)	1.25 (0.91-1.72)	**1.50 (1.21-1.86)**	**1.80 (1.33-2.45)**	**1.94 (1.57-2.41)**	**2.35 (1.73-3.20)**	**2.94 (2.32-3.72)**

Abbreviations: aHR, adjusted hazard ratio; aOR, adjusted odds ratio. Adjusted for recipient BMI, recipient age, recipient sex, donor sex, donor age, donor-recipient weight ratio, DCD status, HLA mismatch, recipient diabetes, PRA group, donor type, cause of ESKD and previous kidney transplant.

Table S2: Adjusted risk of death-censored graft loss for recipient pre-transplant SCr (mg/dL), stratified by recipient median BMI (≤ 27 versus >27 kg/m^2). Reference category used was pre-transplant SCr <5 mg/dL. *Bolded values are statistically significant.*

	Pre-transplant serum creatinine (mg/dL)		
	5-8	8-12	>12
BMI ≤ 27 kg/m^2 (aHR)	1.00 (0.83-1.19)	0.95 (0.79-1.14)	1.09 (0.91-1.32)
BMI > 27 kg/m^2 (aHR)	1.07 (0.89-1.29)	**1.23 (1.02-1.47)**	**1.26 (1.04-1.53)**

Abbreviations: aHR, adjusted hazard ratio. Adjusted for recipient BMI, recipient age, recipient sex, donor sex, donor age, donor-recipient weight ratio, DCD status, HLA mismatch, recipient diabetes, PRA group, donor type, cause of kidney failure and previous kidney transplant.

Table S3: Adjusted risk of death-censored graft loss for recipient pre-transplant SCr (mg/dL), stratified by Kidney Donor Risk Index (KDRI) quartile. Reference category used was pre-transplant SCr <5 mg/dL. This analysis was only applicable to deceased donor transplants. *Bolded values are statistically significant.*

	Pre-transplant serum creatinine (mg/dL)		
	5-8	8-12	>12
KDRI Q1 (≤ 0.916) (aHR)	1.36 (0.92-2.00)	1.19 (0.80-1.76)	1.27 (0.84-1.91)
KDRI Q2 (0.916 - ≤1.108) (aHR)	1.25 (0.87-1.78)	1.30 (0.92-1.85)	1.20 (0.83-1.74)
KDRI Q3 (1.108 – 1.312) (aHR)	0.76 (0.56-1.03)	0.88 (0.65-1.19)	0.79 (0.57-1.09)
KDRI Q4 (>1.312) (aHR)	0.97 (0.72-1.29)	1.17 (0.88-1.55)	**1.36 (1.01-1.84)**

Abbreviations: aHR, adjusted hazard ratio; KDRI, kidney donor risk profile. Adjusted for recipient BMI, recipient age, recipient sex, donor sex, donor age, donor-recipient weight ratio, DCD status, HLA mismatch, recipient diabetes, PRA group, donor type, cause of kidney failure and previous kidney transplant.

Table S4: Adjusted risk of graft outcomes for recipient pre-transplant eGFR (mL/min/1.73 m^2). Reference category used was pre-transplant eGFR >12 mL/min/1.73 m^2. *Bolded values are statistically significant.*

	Pre-transplant eGFR (mL/min/1.73 m^2)		
	<4 (n= 4,083)	4-8 (n= 8,726)	8-12 (n= 4,347)
Death-censored graft loss (aHR)	**1.18 (1.03-1.35)**	1.07 (0.95-1.21)	1.02 (0.89-1.16)
All-cause graft loss (aHR)	1.02 (0.93-1.12)	0.97 (0.89-1.05)	0.96 (0.88-1.05)
Delayed graft function (aOR)	**2.78 (2.31-3.35)**	**1.85 (1.56-2.19)**	**1.38 (1.14-1.66)**

Abbreviations: aHR, adjusted hazard ratio; aOR, adjusted odds ratio. Adjusted for recipient BMI, recipient age, recipient sex, donor sex, donor age, donor-recipient weight ratio, DCD status, HLA mismatch, recipient diabetes, PRA group, donor type, cause of ESKD and previous kidney transplant.

Table S5: Adjusted risk of death-censored graft loss for recipient pre-transplant SCr (mg/dL), excluding patients with graft loss in the first 90 days. Reference category used was pre-transplant SCr <5 mg/dL. *Bolded values are statistically significant.*

	Pre-transplant serum creatinine (mg/dL)		
	5-8	8-12	>12
Death-censored graft loss (aHR)	1.03 (0.90-1.18)	1.10 (0.96-1.26)	**1.19 (1.03-1.37)**

Abbreviations: aHR, adjusted hazard ratio; OR, odds ratio. Adjusted for recipient BMI, recipient age, recipient sex, donor sex, donor age, donor-recipient weight ratio, DCD status, HLA mismatch, recipient diabetes, PRA group, donor type, cause of kidney failure and previous kidney transplant.

Table S6a: Variables selected in multivariable model for the association between pre-transplant serum creatinine and death-censored graft loss.

Variable	aHR	95% CI
Donor age	1.02	1.01-1.02
Recipient age	0.97	0.96-0.97
Donor sex (female)	1.00	0.92-1.08
Recipient sex (female)	1.13	1.05-1.23
Recipient BMI	1.00	1.00-1.01
DR weight mismatch	0.79	0.70-0.89
Recipient DM	1.08	0.90-1.29
Cause of kidney failure		
DM	0.99	0.81-1.21
PCKD	0.71	0.59-0.84
Hypertension	1.13	1.02-1.25
Other	0.87	0.77-0.97
Prior transplant status	1.03	0.91-1.17
Donor type (living)	0.75	0.69-0.81
DCD status	0.97	0.83-1.14
HLA MM		
0	Ref	-
1	1.20	0.95-1.51
2	1.30	1.08-1.56
3	1.35	1.16-1.58

4	1.29	1.11-1.51
5	1.49	1.29-1.74
6	1.55	1.31-1.82
PRA		
<20%	Ref	-
20-80%	1.32	1.18-1.49
>80%	1.36	1.14-1.62

Abbreviations: aHR, adjusted hazard ratio; DR, donor-recipient; BMI, body mass index; DM, diabetes mellitus; PCKD, polycystic kidney disease; DCD, donation after circulatory death; HLA, human leukocyte antigen; MM, mismatch; PRA, peak panel reactive antibody.

Table S6b: Variables selected in multivariable model for the association between pre-transplant serum creatinine and all-cause graft loss.

Variable	aHR	95% CI
Donor age	1.01	1.00-1.01
Recipient age	1.00	1.00-1.01
Donor sex (female)	1.00	0.95-1.06
Recipient sex (female)	0.99	0.94-1.05
Recipient BMI	1.00	0.99-1.00
DR weight mismatch	0.84	0.77-0.92
Recipient DM	1.32	1.18-1.48
Cause of kidney failure		
DM	1.17	1.03-1.33
PCKD	0.67	0.60-0.76
Hypertension	1.13	1.05-1.22
Other	1.10	1.01-1.20
Prior transplant status	1.12	1.02-1.23
Donor type (living)	0.74	0.70-0.79
DCD status	0.92	0.82-1.03
HLA MM		
0	Ref	-
1	1.09	0.93-1.28
2	1.24	1.09-1.40
3	1.25	1.12-1.39

4	1.10	0.99-1.22
5	1.23	1.11-1.36
6	1.23	1.09-1.38
PRA		
<20%	Ref	-
20-80%	1.18	1.08-1.28
>80%	1.13	0.98-1.29

Abbreviations: aHR, adjusted hazard ratio; DR, donor-recipient; BMI, body mass index; DM, diabetes mellitus; PCKD, polycystic kidney disease; DCD, donation after circulatory death; HLA, human leukocyte antigen; MM, mismatch; PRA, peak panel reactive antibody.

Table S6c: Variables selected in multivariable model for the association between pre-transplant serum creatinine and delayed graft function.

Variable	aOR	95% CI
Donor age	1.01	1.01-1.02
Recipient age	1.00	1.00-1.00
Donor sex (female)	0.90	0.81-1.00
Recipient sex (female)	0.81	0.73-0.90
Recipient BMI	1.02	1.01-1.03
DR weight mismatch	1.13	0.96-1.33
Recipient DM	1.34	1.08-1.65
Cause of kidney failure		
DM	1.07	0.85-1.36
PCKD	0.97	0.79-1.19
Hypertension	0.98	0.85-1.13
Other	0.99	0.84-1.16
Prior transplant status	1.04	0.87-1.24
Donor type (living)	0.22	0.19-0.26
DCD status	2.33	2.02-2.68
HLA MM		
0	Ref	-
1	1.06	0.78-1.46
2	0.91	0.71-1.18
3	0.90	0.72-1.10

4	1.00	0.83-1.21
5	1.11	0.92-1.34
6	1.23	1.00-1.52
PRA		
<20%	Ref	-
20-80%	1.13	0.97-1.31
>80%	1.17	0.93-1.47

Abbreviations: aOR, adjusted odds ratio; DR, donor-recipient; BMI, body mass index; DM, diabetes mellitus; PCKD, polycystic kidney disease; DCD, donation after circulatory death; HLA, human leukocyte antigen; MM, mismatch; PRA, peak panel reactive antibody.
